# Acute Exacerbation of Interstitial Lung Disease in Adult Patients With Idiopathic Inflammatory Myopathies: A Retrospective Case-Control Study

**DOI:** 10.3389/fmed.2020.00012

**Published:** 2020-01-31

**Authors:** Junyu Liang, Heng Cao, Yini Ke, Chuanyin Sun, Weiqian Chen, Jin Lin

**Affiliations:** Department of Rheumatology, The First Affiliated Hospital, College of Medicine, Zhejiang University, Hangzhou, China

**Keywords:** interstitial lung disease, dermatomyositis, polymyositis, complication, outcome

## Abstract

**Objective:** This study aimed at clarifying the prevalence, risk factors, outcome, and outcome-related factors of acute exacerbation of interstitial lung disease (AE-ILD) in patients with idiopathic inflammatory myopathy (IIM).

**Methods:** Data of IIM patients who were admitted to the First Affiliated Hospital of Zhejiang University (FAHZJU) from September 2007 to September 2019 were retrospectively collected. And the IIM patients with AE-ILD formed the case group. In addition, age and sex matched IIM patients without AE-ILD were randomly selected to constitute the control group. A 1:2 case-control study and intragroup analysis were performed to identify risk factors for development of AE-ILD in IIM patients and unfavorable short-term outcome in AE-ILD patients through comparison, univariate and multivariate logistic regression analysis.

**Results:** AE-ILD occurred in 64 out of 665 IIM patients (9.6%) with a short-term mortality rate of 39.1%. And the 64 IIM patients with AE-ILD formed the case group. Besides, 128 age and sex matched IIM patients without AE-ILD were randomly selected to constitute the control group. The retrospective case-control study revealed that elevated on-admission disease activity (*P* < 0.001), lower percent-predicted diffusing capacity of the lung for carbon monoxide (DLCO%, *P* = 0.013) and diagnosis of clinically amyopathic dermatomyositis (CADM, *P* = 0.007) were risk factors for development of AE-ILD in IIM patients. The following intragroup analysis indicated that elevated on-admission disease activity (*P* = 0.008) and bacterial infection (*P* = 0.003) were significantly correlated with the unfavorable short-term outcome of patients complicated with AE-ILD. In addition, combined use of steroid and disease modifying antirheumatic drugs (DMARDs, *P* = 0.006) was found to significantly reduce the short-term mortality in IIM patients with AE-ILD.

**Conclusion:** AE-ILD is a less frequent but fatal complication in IIM patients with elevated on-admission disease activity, lower DLCO% and diagnosis of CADM working as risk factors, indicating the potential roles of autoimmune abnormality and hypoxia in development of AE-ILD. Elevated on-admission disease activity and bacterial infection could predict unfavorable short-term outcome of IIM patients with AE-ILD. A therapeutic regimen of steroid and DMARDs was found to reduce short-term death in these patients.

## Introduction

Idiopathic inflammatory myopathies (IIM) are a group of autoimmune diseases that primarily target the skeleton muscles (1, 2). Dermatomyositis (DM) and polymyositis (PM) are two conventional subtypes of IIM, while clinically amyopathic dermatomyositis (CADM) is a newly recognized subset of DM with typical skin rash of DM and slight muscular damage. Although the incidence of DM, PM, and CADM was considerably low in common people, the high mortality rate, the various clinical manifestations, and multiple complications have drawn much attention from clinicians and researchers. In published studies, the 10-year survival rate for patients with DM, PM, or CADM ranged from 51 to 91% (3). An ~4.5% in-hospital mortality rate was seen in two retrospective studies (3, 4).

Multiple organs apart from muscle are often affected as well, leading to critical worsening of the life quality and outcome of these patients (5). Among the multiple extramuscular complications of IIM, interstitial lung disease (ILD) was identified as both the most frequent and severe involvement, leading to a significant elevation in mortality rate (6). Moreover, acute exacerbation of ILD (AE-ILD), which used to be mainly studied in patients with idiopathic pulmonary fibrosis, has also been noticed in patients with connective tissue disease (CTD). In CTD patients, AE-ILD was reported to occur at a 1-year frequency of 1.25–3.3%, at a lifetime incidence of 7.2% in CTD patients, and contributed to a high mortality rate within these patients (7, 8). In the past few years, there existed a few reports and small-sample studies of AE-ILD, or rapid progression of ILD, in IIM patients. However, systemic understandings including the incidence of AE-ILD, its risk factors and outcome in IIM patients remained unclear. It is thus necessary to uncover the enigma by figuring out factors correlated with AE-ILD in patients with DM, PM, or CADM, and factors associated with outcome of patients with AE-ILD.

In this study, we retrospectively reviewed the medical records of 424 patients with DM, PM, and CADM who were admitted to our center from February 2011 to February 2019, and performed a case-control analysis to identify potential related risk factors for AE-ILD among these patients. Besides, factors affecting the short-term outcome of patients with AE-ILD were as well-probed into via subgroup analysis.

## Materials and Methods

### Patients

Medical records of adult patients who were admitted to the inpatient department of the Qingchun division of the First Affiliated Hospital of Zhejiang University (FAHZJU) with the diagnosis of DM, PM, or CADM from September 2007 to September 2019 was reviewed and collected. The approval (Reference Number: 2019-646) of the Institutional Review Board (IRB) of the FAHZJU was acquired before the initiation of the study, and written informed consent from each patient involved was acquired as well. The inclusion criteria of this study were: (1) age over 18 years old; (2) the diagnosis of DM or PM fulfilled the diagnostic criteria of Bohan and Peter (9), and the diagnosis of CADM met the criteria developed by Sontheimer (10). Exclusion criteria were: (1) overlap syndromes with other connective tissue diseases; (2) hospitalization for causes unrelated to myositis and its complications, such as fracture, pregnancy, cataract, and appendicitis etc.; (3) myopathies that might be related to thyroid dysfunction, excessive exercises, inherited, or metabolic disorders, recent use of muscle-impairment drugs including statins, chloroquine, colchicine, entecavir, traditional Chinese medicine, etc.; (4) loss to follow-up within 2 weeks after discharge.

### Methods

Medical records of all patients enrolled were retrospectively collected by reviewing the electronic medical record (EMR) system. Data including demographic information, course of disease, duration of diagnosis delay, clinical manifestations, or complications, on-admission disease activity, results of pulmonary function test, preceding comorbidities, harmful hobbies, imaging reports, laboratory findings, medications, as well as short-term outcome were acquired and analyzed. ILD, subtype of ILD and AE-ILD were evaluated by radiologists using high-resolution computed tomography (HRCT). In absence of diagnostic criteria dedicated to AE-ILD in patients with CTD, an updated criteria of acute exacerbation of idiopathic pulmonary fibrosis (AE-IPF) was adopted based on the experience of published studies on AE-ILD in CTD patients. The updated criteria included previous or concurrent diagnosis of ILD, acute worsening or development of dyspnea typically <1 month duration, computed tomography with new bilateral ground-glass opacity and/or consolidation superimposed on a background pattern consistent with usual interstitial pneumonia (UIP) pattern, and deterioration not fully explained by cardiac failure or fluid overload (11). Compared with the previous diagnostic criteria for AE-ILD proposed in 2007 (12), the new criteria does not demand thorough exclusion of infection. And infection has been found to participate in the pathogenesis and progression of idiopathic pulmonary fibrosis (IPF) (13). As previously suggested, the occurrence of this clinical and radiological manifestation in a background of possible or inconsistent with UIP pattern was also considered diagnostic for AE in CTD patients (14, 15). Cases manifested as UIP pattern were identified based on their radiologic appearance on HRCT: the presence of basal-dominant reticular opacities and predominantly basal and subpleural distribution of honeycomb lesions, with multiple equal-sized cystic lesions of 2–10 mm diameter with a thick wall (16). Diagnosis of bacterial, fungal, or tuberculosis infection was a comprehensive decision based on the essential positive result of etiological detection, HRCT manifestation, clinical symptoms, infection-related laboratory abnormalities, treatment of intravenous antibiotics, and antifungal drugs, positive response after treatment, etc. The etiological detection was defined as the culture of bronchoalveolar lavage fluid (BALF) and sputum. Sputum culture result counted only if >25 squamous epithelial cells per low-power field were observed (17). In bacterial infections, the thresholds for positivity of quantitative cultures were applied: 10^5^ cfu/ml for sputum culture (17), 10^4^ cfu/mL for bronchoalveolar lavage (18). For patients with infection of *Candida albican* or *Candida glabrata*, the BALF or sputum culture should show a visually medium to large amount of *C. albicans* or *C. glabrata* in the sample. The repeated cultures of BALF or sputum were routinely initiated before intravenous use of antibiotics or anti-fungal medications. Meanwhile diagnosis of virus infection, to be specific, Epstein-Barr virus (EBV) or Cytomegalo virus (CMV) infection, relied on the screening of serum antibody and DNA of these two viruses. Identification of gastrointestinal hemorrhage was based on repeated positive results of fecal occult blood test. To minimize omission of lymphadenectasis, hepatomegaly, and splenomegaly, the identification was based on records of physical examination together with reports of ultrasound examination, computed tomography and positron emission tomography. On-admission disease activity was routinely assessed by the Myositis Disease Activity Assessment Visual Analog Scales (MYOACT) within the first week of admission (19). Immunosuppressive regimens used during hospitalization were categorized into four groups: (1) steroid monotherapy; (2) steroid + disease-modifying antirheumatic drugs (DMARDs); (3) steroid + intravenous immunoglobulin (IVIG); (4) steroid + DMARDs +IVIG. In this study, usage of DMARDs included usage of mycophenolate mofetil (MMF), thalidomide, hydroxychloroquine, cyclosporine, azathioprine, methotrexate, cyclophosphamide, etc. Short-term mortality, or unfavorable short-term outcome, referred to in-hospital mortality or death within 2 weeks of hospital discharge.

To probe into factors exerting significant influence on development of AE-ILD within patients with DM, PM, or CADM, a case-control study was performed. Patients diagnosed with AE-ILD constituted the case group. And ILD patients without AE-ILD were selected using a systematic sampling method by matching age and sex with cases with AE-ILD at a proportion of 1:2. Comparisons, univariate and multivariate logistic regression analysis were performed between the case group and the control group. To clarify the time axis of risk factors and results, only clinical manifestations or complications that happened before the diagnosis of AE-ILD would be taken into account for patients with AE-ILD. In order to identity potential factors affecting the short-term outcome of the AE-ILD patients involved, the AE-ILD patients were further divided into two groups: patients who died in hospital or within 2 weeks of hospital discharge were defined as the mortality group, and those who survived after 2 weeks of hospital discharge were categorized as the survival group. Comparisons and logistic regression analysis were made between the two groups of patients regarding age, sex, clinical features, disease activity, laboratory findings, etc.

### Statistical Analysis

Statistical analysis was performed using SPSS 22.0 (Chicago, IL, USA) and R 3.6.1. The normality of continuous variables was tested by the Kolmogorov-Smirnov goodness-of-fit model. Continuous variables were expressed as mean ± SD if normally distributed and median (quartiles) if skewed. Ordinal categorical variables were as well shown as median (quartiles). Unordered categorical variables were presented as numbers and percentages. Independent sample *t*-test was used to compare normally distributed continuous variables. And Mann-Whitney *U*-test was applied to compare skewed continuous variables or ordinal categorical variables. Chi-square test and Fisher's exact test were used to compare unordered categorical variables. All tests were two-sided and a *P* < 0.05 was considered statistically significant. Univariate and multivariate logistic regression analyses were subsequently adopted to identify risk factors for AE-ILD in patients with PM, DM or CADM as well as risk factors for unfavorable short-term outcome in AE-ILD. In the study of risk factors for AE-ILD, explanatory factors with *P* < 0.1 in the univariate logistic regression analysis were entered into the multivariate logistic regression analysis. In the process of figuring out risk factors for unfavorable short-term outcome, however, factors with *P* < 0.05 in univariate analysis were enrolled into the multivariate logistic regression analysis owing to the limited number of AE-ILD patients. For normally distributed continuous variables with missing values, inputation using expectation maximization (EM) algorithm was performed for those that passed univariate screening. Multivariate logistic regression analysis with a stepwise forward likelihood ratio (LR) method was used to determine the statistically significant factors. Results from the multivariate logistic regression were presented as an odds ratio (OR) with 95% confidence interval (CI). A two-sided *P* < 0.05 was considered to be statistically significant. If there existed any positive result in serum biomarkers or disease activity in multivariate logistic regression analysis, a receiver operating characteristic (ROC) curve analysis would be performed to evaluate its predictive value for development and outcome of AE-ILD.

## Results

**Figure 1:**
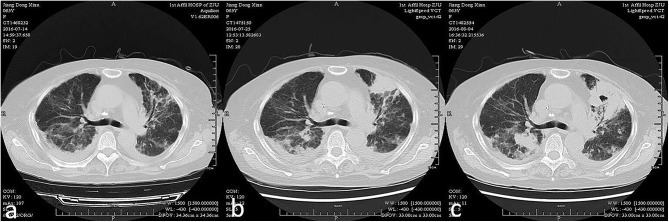
Acute exacerbation of interstitial lung disease of a patient within 3 weeks (from **a–c** chronologically).

A total of 665 patients treated at FAHZJU with a diagnosis of DM, PM, or CADM between September 2007 and September 2019 were enrolled into this study, including 334 with DM, 264 with PM, and 67 with CADM. Four hundred and eighty-three patients (72.6%) were identified to be complicated with ILD. Sixty-four out of 665 patients were diagnosed with AE-ILD during their stay in hospital (Figure 1). The incidence of AE-ILD was 9.6% in patients with DM, PM, or CADM, and 13.3% in patients who were complicated with ILD at the same time. To be specific, the incidence of AE-ILD in patient with DM, PM, and CADM were 10.8, 5.7, and 19.4%, respectively. In the 665 patients, the average age for AE-ILD patients was 57.7 ± 11.9 years, which was significantly higher than that of the patients without AE-ILD (53.1 ± 13.7 years, *P* = 0.011). Among the 64 AE-ILD patients, 25 were males and 39 were females. The proportion of males in AE-ILD patients was not significantly different from that in non-AE-ILD patients (39.1 vs. 32.3%, *P* = 0.272). Short-term mortality rate for AE-ILD and non-AE-ILD patients were 39.1 vs. 5.7% (*P* < 0.001).

In total, 64 AE-ILD patients and 128 ILD patients without occurrence of AE-ILD were included in the case-control analysis to identify risk factors for AE-ILD in patients with DM, PM, or CADM. Due to the retrospective nature of this study, only 137 patients (54 of AE-ILD patients and 83 of patients without AE-ILD) received pulmonary function test within the first week of hospitalization. The case group presented more frequently with treatment of steroid + IVIG (*P* = 0.034), diagnosis of CADM (*P* = 0.034) and less frequently with allergic history (*P* = 0.049). Higher levels of serum ferritin (*P* = 0.027) and C reactive protein (CRP, *P* = 0.004) were seen in patients with AE-ILD. On-admission disease activity, which was evaluated by MYOACT score, was as well-significantly higher for patients in the case group (*P* < 0.001). In addition, AE-ILD patients were found to present with lower level of percent-predicted diffusing capacity of the lung for carbon monoxide (DLCO%, *P* = 0.009; Table 1, Supplementary Data 1).

**Table 1:** Comparison of clinical characteristics between case group and control group.

**Factors**	**AE-ILD (64)**	**Non-AE-ILD (128)**	***P*-value**
Age (y)	60.5 (48.0, 66.0)	60.0 (48.3, 65.0)	0.726
Sex (male/female)	25/39	50/78	1.000
Course of disease (m)	3.0 (1.0, 6.8)	4.0 (2.0, 8.8)	0.122
Duration of diagnosis delay (m)	2.0 (1.0, 4.5)	3.0 (1.0, 6.0)	0.113
**Clinical manifestations or complications**			
Fever	27 (42.2%)	40 (31.3%)	0.134
Lymphadenectasis	26 (40.6%)	47 (36.7%)	0.599
Hepatomegaly	1 (1.6%)	1 (0.8%)	1.000
Splenomegaly	14 (21.9%)	21 (16.4%)	0.355
Heliotrope rash	33 (51.6%)	63 (49.2%)	0.759
Gottron's sign	36 (56.3%)	65 (50.8%)	0.474
Periungual erythema	13 (20.3%)	21 (16.4%)	0.504
Mechanic's hands	9 (14.1%)	17 (13.3%)	0.881
Raynaud's phenomenon	4 (9.5%)	8 (9.5%)	1.000
Muscle pain	22 (34.4%)	53 (41.4%)	0.347
Muscle weakness	50 (78.1%)	111 (86.7%)	0.127
Joint pain	17 (26.6%)	24 (18.8%)	0.213
Joint swelling	8 (12.5%)	21 (16.4%)	0.476
Dysphagia	11 (17.2%)	27 (21.1%)	0.522
Dysarthria	5 (7.8%)	8 (6.3%)	0.919
Respiratory muscle involvement	2 (3.1%)	7 (5.5%)	0.717
Cardiac involvement	4 (6.3%)	10 (7.8%)	0.922
Gastrointestinal hemorrhage	9 (14.1%)	15 (11.7%)	0.643
Bacterial infection	14 (21.9%)	21 (16.4%)	0.355
Fungal infection	15 (23.4%)	22 (17.2%)	0.301
Tuberculosis infection	3 (4.7%)	3 (2.3%)	0.402
EBV or CMV infection	2 (3.1%)	6 (4.7%)	0.890
Carcinoma	6 (9.4%)	11 (8.6%)	0.857
UIP pattern	15 (23.4%)	23 (18.0%)	0.370
Pneumomediastinum	4 (6.3%)	6 (4.7%)	0.909
**On-admission disease activity**			
MYOACT score	10.0 (8.0,12.0)	7.0 (5.0,9.0)	<0.001
**Pulmonary function test**			
FVC% (%)	66.1 ± 17.9	67.4 ± 19.2	0.684
TLC (L)	3.1 (2.6,4.3)	3.6 (2.9,4.2)	0.107
FEV1% (%)	66.8 ± 15.8	70.4 ± 21.3	0.288
FEV1/FVC	0.8 (0.7,0.9)	0.8 (0.8,0.9)	0.335
DLCO% (%)	53.6 ± 15.4	62.3 ± 20.5	0.009
**On-admission laboratory findings**			
ALT (U/L)	49.0 (22.8,122.3)	50.0 (27.0,134.0)	0.710
AST (U/L)	48.0 (29.5,105.8)	61.5 (31.5,163.3)	0.283
Cr (μmol/L)	52.0 (43.0,69.0)	49.5 (43.0,59.0)	0.129
LDH (U/L)	421.0 (330.8,619.3)	401.0 (300.5,820.8)	0.844
CK (U/L)	179.0 (54.3,958.5)	484.5 (58.0,2465.5)	0.113
CK-MB (U/L)	31.5 (18.3,55.5)	32.0 (19.0,110.0)	0.210
CRP (mg/L)	10.1 (4.5,43.7)	6.1 (2.3,18.8)	0.004
Ferritin (ng/ml)	821.7 (342.9,2034.5)	532.7 (247.4,1205.9)	0.027
ANA	40 (62.5%)	75 (58.6%)	0.603
**Comorbidities/Harmful hobbies**			
Smoking	14 (21.9%)	26 (20.3%)	0.802
Alcohol abuse	10 (15.6%)	24 (18.8%)	0.593
Hypertension	22 (34.4%)	28 (21.9%)	0.063
Diabetes	8 (12.5%)	12 (9.4%)	0.504
Hepatitis	4 (6.3%)	15 (11.7%)	0.232
Allergic History	4 (6.3%)	21 (16.4%)	0.049
**Immunosuppressive therapy**			
Steroid monotherapy	19 (29.7%)	37 (28.9%)	0.911
Steroid + DMARDs	29 (45.3%)	71 (55.5%)	0.184
Steroid + IVIG	13 (20.3%)	12 (9.4%)	0.034
Steroid + DMARDs + IVIG	3 (4.7%)	8 (6.3%)	0.913
**IIM subtypes**			
DM	36 (56.3%)	72 (56.3%)	1.000
PM	15 (23.4%)	44 (34.4%)	0.122
CADM	13 (20.3%)	12 (9.4%)	0.034

*AE-ILD, Acute exacerbation of interstitial lung disease; y, years; m, months; EBV, Epstein-Barr virus; CMV, Cytomegalo virus; UIP pattern, Usual interstitial pneumonia pattern; MYOACT, Myositis Disease Activity Assessment Visual Analog Scales; FVC%, Percent-predicted forced vital capacity; TLC, Total lung capacity; FEV1%, Percent-predicted forced expiratory volume in 1 s; FEV1/FVC, Ratio of FEV1 over FVC; DLCO%, Percent-predicted diffusing capacity of the lung for carbon monoxide; ALT, Glutamic pyruvic transaminase; AST, Glutamic oxaloacetic transaminase; Cr, Serum creatinine; LDH, Lactate dehydrogenase; CK, Creatine kinase; CK-MB, Creatine kinase isoenzymes; ANA, Antinuclear antibody; DMARDs, Disease-modifying anti-rheumatic drugs; IVIG, Intravenous immunoglobulin; IIM, Idiopathic inflammatory myopathies; DM, dermatomyositis; PM, Polymyositis; CADM, Clinically amyopathic dermatomyositis*.

**Figure 2:**
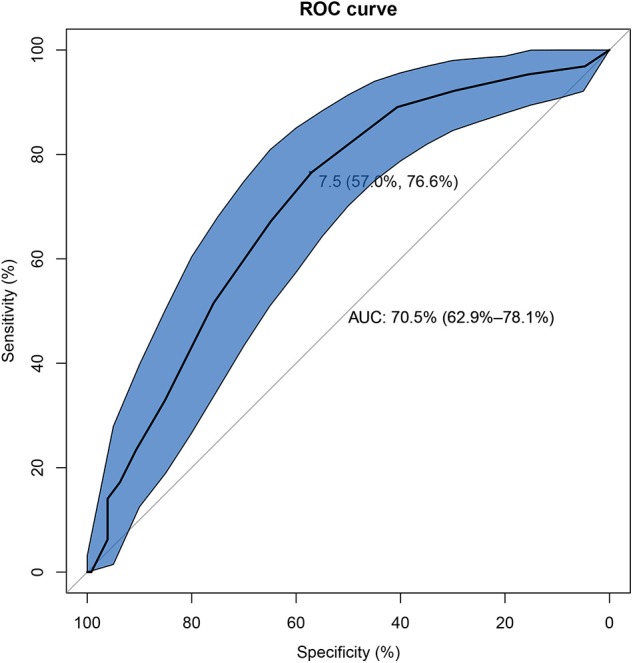
The receiver operating characteristic curve of on-admission disease activity for development of AE-ILD in IIM patients. AE-ILD, Acute exacerbation of interstitial lung disease; IIM, Idiopathic inflammatory myopathies.

Univariate analysis showed that there were eight factors associated with AE-ILD at the level of *P* < 0.1. These factors included elevated on-admission disease activity (*P* < 0.001), lower DLCO% (*P* = 0.010), serum ferritin (*P* = 0.058), CRP (*P* = 0.037), hypertension (*P* = 0.065), allergic history (*P* = 0.058), treatment of steroid + IVIG (*P* = 0.038), and diagnosis of CADM (*P* = 0.038) (Supplementary Table 1). Inputation was performed for DLCO% before multivariate logistic regression analysis. Using Kolmogorov-Smirnov test, DLCO% was found a continuous variable that was subject to normal distribution. EM inputation was hereby performed to handle the impact of missing values more appropriately. Afterwards, all variables with *P* < 0.1 were entered into the multivariate logistic regression analysis, and elevated on-admission disease activity (*P* < 0.001), lower DLCO% (*P* = 0.013), and diagnosis of CADM (*P* = 0.007) were found to be significantly different between the case group and the control group. The results were found similar to those without EM imputation (Table 2). As presented in Figure 2, the optimal cut-off value of the on-admission disease activity for AE-ILD was >7.5, with a sensitivity of 76.6% and a specificity of 57.0%. The area under the curve (AUC) was 0.705.

**Table 2:** Multivariate logistic regression analysis of risk factors for AE-ILD in patients with DM, PM, or CADM.

**Factors**	***P-*value**	**OR value**	**95% Cl**
On-admission disease activity (MYOACT score)	<0.001	1.243	1.127–1.371
DLCO%	0.013	0.972	0.950–0.994
CADM	0.007	3.781	1.444–9.903

*DM, dermatomyositis; PM, Polymyositis; CADM, Clinically amyopathic dermatomyositis; OR value, Odds ratio value; 95%Cl, 95% Confidence interval; MYOACT, Myositis Disease Activity Assessment Visual Analog Scales, DLCO%, Percent-predicted diffusing capacity of the lung for carbon monoxide*.

Of the 64 AE-ILD patients identified in the study, 25 (39.1%) died in hospital or within 2 weeks of hospital discharge. In addition to 36 AE-ILD patients with DM, we also found 15 PM patients and 13 CADM patients who as well-suffered from AE-ILD. And 15 of them (23.4%) manifested as UIP pattern in HRCT. Infection happened to 30 out of 64 adult AE-ILD patients. Ten had bacterial infection, 12 had fungal infection, three were diagnosed with tuberculosis, one was found to have EBV infection. Three suffered from both bacterial and fungal infection, and one had both bacterial and EBV infection. Bacterial (21.9%) and fungal (23.4%) infections were hereby recognized as the two most common infections in AE-ILD patients. Only eight patients with infections (five in bacterial infection, two in fungal infection, and one in tuberculosis infection) were identified based on positive result of BALF smear or culture. To be specific, bacterial infection included four cases of *Acinetobacter baumannii*, four cases with *Stenotrophomonas maltophilia*, three case with *Klebsiella pneumonia*, onc case with *Pseudomonas* aeruginosa, one case with *Staphylococcus haemolyticus*, and one case with *Staphylococcus aureus*. And fungal infection included 10 cases with medium to large amount of *C. albicans*, three cases with *Aspergillus fumigatus*, one case with *Pneumocystis carinii* and one case with *C. glabrata*. Therefore, infections in patients with AE-ILD were mostly opportunistic infections. Details on infections in the matched control group was provided in Supplementary Data 2. In addition, the most commonly used therapy was a combined application of steroid and DMARDs (45.3%). And MMF (48.3%) was the most frequently used DMARD in this regimen. Patients with unfavorable short-term outcome presented more frequently with dysphagia (*P* = 0.030), bacterial infection (*P* = 0.001), hypertension (*P* = 0.017), treatment of steroid + IVIG (*P* = 0.013), and less frequently with treatment of steroid + DMARDs (*P* = 0.001). Higher on-admission disease activity (*P* = 0.014) was as well-seen in patients with unfavorable outcome (Table 3).

**Table 3:** Comparison of clinical characteristics between mortality group and survival group.

**Factors**	**Mortality group (25)**	**Survival group (39)**	***P*-value**
Age (y)	62.0 (47.0,67.0)	60.0 (51.0,65.0)	0.967
Sex (male/female)	12/13	13/26	0.241
Course of disease (m)	2.0 (1.0,4.5)	3.0 (1.0,9.0)	0.235
Duration of diagnosis delay (m)	2.0 (1.0,3.0)	3.0 (1.0,6.0)	0.332
**Clinical manifestations or complications**			
Fever	14 (56.0%)	13 (33.3%)	0.073
Lymphadenectasis	8 (32.0%)	18 (46.2%)	0.261
Hepatomegaly	1 (4.0%)	0 (0.0%)	0.391
Splenomegaly	6 (24.0%)	8 (20.5%)	0.742
Heliotrope rash	12 (48.0%)	21 (53.8%)	0.648
Gottron's sign	12 (48.0%)	24 (61.5%)	0.287
Periungual erythema	4 (16.0%)	9 (23.1%)	0.492
Mechanic's hands	4 (16.0%)	5 (12.8%)	1.000
Raynaud's phenomenon	0 (0.0%)	4 (10.3%)	0.149
Muscle pain	11 (44.0%)	11 (28.2%)	0.194
Muscle weakness	20 (80.0%)	30 (76.9%)	0.771
Joint pain	7 (28.0%)	10 (25.6%)	0.835
Joint swelling	3 (12.0%)	5 (12.8%)	1.000
Dysphagia	8 (32.0%)	3 (7.7%)	0.030
Dysarthria	4 (16.0%)	1 (2.6%)	0.072
Respiratory muscle involvement	1 (4.0%)	1 (2.6%)	1.000
Cardiac involvement	3 (12.0%)	1 (2.6%)	0.291
Gastrointestinal hemorrhage	5 (20.0%)	4 (10.3%)	0.468
Bacterial infection	11 (44.0%)	3 (7.7%)	0.001
Fungal infection	9 (36.0%)	6 (15.4%)	0.057
Tuberculosis infection	0 (0.0%)	3 (7.7%)	0.275
EBV or CMV infection	0 (0.0%)	2 (5.1%)	0.516
Carcinoma	0 (0.0%)	6 (15.4%)	0.074
UIP pattern	5 (20.0%)	10 (25.6%)	0.603
Pneumomediastinum	3 (12.0%)	1 (2.6%)	0.291
**On-admission disease activity**			
MYOACT score	10.0 (9.0, 14.5)	9.0 (7.0, 12.0)	0.014
**Pulmonary function test**			
FVC% (%)	61.7 (36.7, 85.1)	69.0 (58.8, 79.3)	0.248
TLC (L)	3.2 (2.6, 4.3)	3.1 (2.4, 4.4)	0.787
FEV1% (%)	64.0 (42.7, 77.2)	69.4 (60.9, 78.6)	0.205
FEV1/FVC	0.8 (0.7,0.9)	0.8 (0.7,0.9)	0.615
DLCO% (%)	51.1 (44.9, 61.8)	58.2 (42.8, 63.2)	0.533
**On-admission laboratory findings**			
ALT (U/L)	63.0 (29.5, 120.5)	39.0 (21.0, 139.0)	0.559
AST (U/L)	60.0 (34.5, 97.0)	44.0 (24.0, 215.0)	0.461
Cr (μmol/L)	67.0 (41.0, 98.0)	52.0 (43.0, 63.0)	0.198
LDH (U/L)	439.0 (369.0, 609.5)	403.0 (317.0, 625.0)	0.518
CK (U/L)	151.0 (38.0, 312.0)	193.0 (93.0, 1667.0)	0.128
CK-MB (U/L)	25.0 (17.0, 58.5)	37.0 (20.0, 54.0)	0.405
CRP (mg/L)	18.7 (5.4, 53.2)	9.5 (4.4, 27.2)	0.259
Ferritin (ng/ml)	834.9 (611.0, 2757.4)	811.6 (186.4, 1690.2)	0.139
ANA	12 (48.0%)	28 (71.8%)	0.055
**Comorbidities/Harmful hobbies**			
Smoking	6 (24.0%)	8 (20.5%)	0.742
Alcohol abuse	4 (16.0%)	6 (15.4%)	1.000
Hypertension	13 (52.0%)	9 (23.1%)	0.017
Diabetes	4 (16.0%)	4 (10.3%)	0.701
Hepatitis	2 (8.0%)	2 (5.1%)	0.640
Allergic History	2 (8.0%)	2 (5.1%)	0.640
**Immunosuppressive therapy**			
Steroid monotherapy	8 (32.0%)	11 (28.2%)	0.746
Steroid + DMARDs	5 (20.0%)	24 (61.5%)	0.001
Steroid + IVIG	9 (36.0%)	4 (10.3%)	0.013
Steroid + DMARDs + IVIG	3 (12.0%)	0 (0.0%)	0.055
**IIM subtypes**			
DM	14 (56.0%)	22 (56.4%)	0.974
PM	7 (28.0%)	8 (20.5%)	0.490
CADM	4 (16.0%)	9 (23.1%)	0.960

*y, years; m, months; EBV, Epstein-Barr virus; CMV, Cytomegalo virus; UIP pattern, Usual interstitial pneumonia pattern; MYOACT, Myositis Disease Activity Assessment Visual Analog Scales; FVC%, Percent-predicted forced vital capacity; TLC, Total lung capacity; FEV1%, Percent-predicted forced expiratory volume in 1 s; FEV1/FVC, Ratio of FEV1 over FVC; DLCO%, Percent-predicted diffusing capacity of the lung for carbon monoxide; ALT, Glutamic pyruvic transaminase; AST, Glutamic oxaloacetic transaminase; Cr, Serum creatinine; LDH, Lactate dehydrogenase; CK, Creatine kinase; CK-MB, Creatine kinase isoenzymes; ANA, Antinuclear antibody; DMARDs, Disease-modifying anti-rheumatic drugs; IVIG, Intravenous immunoglobulin; IIM, Idiopathic inflammatory myopathies; DM, dermatomyositis; PM, Polymyositis; CADM, Clinically amyopathic dermatomyositis*.

**Figure 3:**
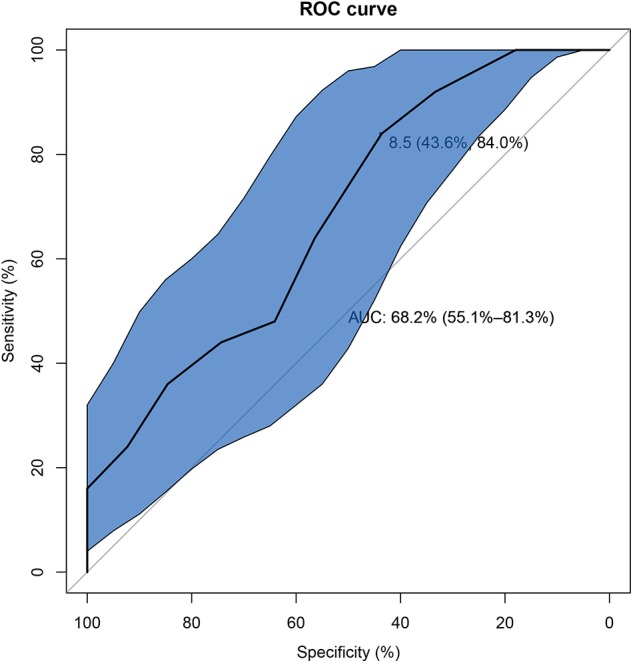
The receiver operating characteristic curve of on-admission disease activity for unfavorable short-term outcome in IIM patients with AE-ILD. IIM, Idiopathic inflammatory myopathies; AE-ILD, Acute exacerbation of interstitial lung disease.

Univariate analysis showed that there were six factors associated with unfavorable short-term outcome in AE-ILD patients at the level of *P* < 0.05. These factors included dysphagia (*P* = 0.019) bacterial infection (*P* = 0.002), on-admission disease activity (*P* = 0.012), hypertension (*P* = 0.020), treatment of steroid + DMARDs (*P* = 0.002) and steroid + IVIG (*P* = 0.018) (Supplementary Table 2). The following multivariate logistic regression analysis revealed that higher on-admission disease activity (*P* = 0.008), bacterial infection (*P* = 0.003), and treatment of steroid+DMARDs (*P* = 0.006) were significantly correlated with unfavorable short-term outcome in AE-ILD patients (Table 4). As presented in Figure 3, the best cut-off value of the on-admission disease activity for unfavorable short-term outcome in patients with AE-ILD was >8.5, with a sensitivity of 84.0% and a specificity of 43.6%. The AUC was 0.682.

**Table 4:** Multivariate logistic regression analysis of risk factors for unfavorable short-term outcome in patients complicated with AE-ILD.

**Factors**	***P*-value**	**OR value**	**95% Cl**
On-admission disease activity (MYOACT score)	0.008	1.346	1.082–1.674
Bacterial infection	0.003	13.494	2.398–75.945
Steroid+DMARDs	0.006	0.137	0.033–0.565

*AE-ILD, Acute exacerbation of interstitial lung disease; MYOACT, Myositis Disease Activity Assessment Visual Analog Scales; DMARDs, Disease-modifying anti-rheumatic drugs*.

## Discussion

To date, this is the first study to systematically probe into the risk factors for development of AE-ILD in patients with DM, PM, or CADM, and potential factors affecting the short-term outcome of the AE-ILD patients. Preceding studies on acute exacerbation mainly focused on AE-IPF. And the annual incidence of AE-IPF ranged from 7 to 19.1% in different clinical trials and retrospective studies (20–25). Knowledge on AE-ILD in non-IPF patients, namely connective-tissue-disease-related ILD (CTD-ILD), was limited. The reported incidence of AE-ILD in rheumatoid arthritis (RA) patients with ILD was 7.7–22% (26, 27). Tomiyama et al. revealed an AE-ILD incidence of 9.4% in systemic sclerosis (28). In this study, the incidence of AE-ILD was 9.6% in patients with DM, PM, or CADM, and 13.3% in patients complicated with ILD. And the mortality rate of AE-ILD was significantly higher than that in non-AE-ILD patients (39.1 vs. 5.7% *P* < 0.001). Besides, the average age for AE-ILD patients was as well-higher than that of the patients without AE-ILD (57.7 ± 11.9 vs. 53.1 ± 13.7 years, *P* = 0.011). Elevated on-admission disease activity, lower DLCO% and diagnosis of CADM were found to be risk factors for development of AE-ILD in patients with DM, PM, or CADM. Moreover, bacterial infection, elevated on-admission disease activity and treatment of steroid + DMARDs were significantly correlated with short-term outcome in AE-ILD patients.

Previous studies revealed that declined forced vital capacity (FVC), low diffusing capacity of the lung for carbon monoxide (DLCO), pulmonary hypertension, comorbid coronary artery disease, surgical resection of lung cancers and various infections etc. were found to be risk factors for AE-ILD (29–31). However, the results were not homogeneous in different studies. In this study, decreased DLCO%, which reflected lower diffusing capacity, was found to be a risk factor for AE-ILD in patients with DM, PM, or CADM. The role of lower DLCO% in AE-ILD was not clear. On the one hand, lower DLCO% reflected decreased gas-exchanging function of lung. With no significant alteration in pulmonary ventilation function etc., decreased gas-exchanging function would lead to hypoxia, which could subsequently contribute to progress of ILD. Hypoxia have been recognized to induce progress of interstitial lung disease through augmenting oxidative and inflammatory pathways, increasing the total lung collagen content and heterogeneous structural alterations (32–34). On the other hand, decreased DLCO% could be an early-stage manifestation of AE-ILD since ILD and its progression could result in impaired diffuse capacity via alveolar structural alteration, thickening of alveolar capillary wall, etc. Lower DLCO% seemed to be both initiating factor and consequence of AE-ILD.

MYOACT score works as a systemic evaluation of disease activity of IIM (19, 35). After adjusting for other factors, elevated on-admission MYOACT score was found to be related to development of AE-ILD in IIM patients. The role of CTD disease activity in AE-ILD was disputable in published studies. In a retrospective study concerning RA patients receiving tocilizumab treatment, AE-ILD was found to be positively related to disease activity of RA (36). However, no similar association was seen in RA patients treated by corticosteroids and immunosuppressants. The predictive role of MYOACT score in this study might lie in the partially overlapped pathological mechanism between AE-ILD and IIM. Elevated levels of several cytokines and chemokines, namely IL-6, IL-8, IL-17, IL-23, etc., were seen in peripheral blood, muscle or skin of IIM patients, and were consistent with disease activity (37). Meanwhile several studies also observed significant elevation of cytokines and chemokines including IL-6, IL-8 in patients with ILD exacerbation, and the elevation was found to be related to worse outcome (38, 39). The partially overlapped pathological mechanism made baseline disease activity a valuable predictor of AE-ILD. Besides, after adjusting for factors including infections, medication, pulmonary function, etc., the significance of on-admission disease activity could, to some extent, demonstrated the role of autoimmune abnormality in development of AE-ILD. In 2011, Shu etc. found that initial disease activity, which was evaluated by MYOACT score, was not significantly correlated with long-term outcome of IIM patients (40). And no linkage between initial disease activity and short-term outcome of hospitalized IIM patients was reported previously. By narrowing down to DM, PM, or CADM patients complicated with AE-ILD, on-admission disease activity, which was evaluated by MYOACT score, was found to herald unfavorable short-term outcome in this study.

However, the evaluation of disease activity demands ability for communication, which would be difficult in patients with mental retardation or disturbed behavior. It would thus be of great significance to identify serum biomarkers for development and outcome of AE-ILD in IIM patients. Researchers in Hamamatsu University found that higher levels of ferritin predicted development of AE-IPF and unfavorable outcome (41). However, in this study, serum ferritin was not found to be significantly related to development of AE-ILD after adjusting for other clinical features. Nor was it identified to predict short-term outcome of IIM patients with AE-ILD. Preceding study also revealed that CRP could be used to predict development of AE-ILD in patients receiving non-pulmonary surgery (42). Nevertheless, no statistical significance for CRP was seen in IIM patients with regard to development and outcome of AE-ILD. Further studies would be demanded to identify serum biomarkers for development and outcome of AE-ILD in CTD patients.

In addition to the high prevalence of ILD in CADM patients, preceding studies proposed that rapidly progressive pattern of ILD was more frequently seen in CADM patients compared with patients with DM or PM (43, 44). After multivariate logistic regression analysis, diagnosis of CADM was found to be a risk factor for AE-ILD in patients with DM, PM or CADM, which was consistent with the past clinical findings. Although CD8+ T cells were found to play a key role in development of IIM-related ILD, high proportion of CD4+ T cells seemed to play a greater role in acute exacerbation of ILD. Suda and his colleagues focused on CADM patients and found that the CD4/CD8 ratio in bronchoalveolar lavage fluid (BALF) was higher in patients with rapidly progressive ILD in comparison to that in chronic ILD patients (45). Ito et al. demonstrated similar results in BALF and peripheral blood of patients with DM (46). Moreover, Mukae et al. uncovered a higher CD4/CD8 ratio in BALF of CADM-related ILD patients compared with that in ILD patients with classic DM (43). Taken together, the higher proportion of CD4+ T cells in BALF seem to link diagnosis of CADM with higher incidence of AE-ILD. Confirmation of the role of higher proportion of CD4+ T cells and exploration of its detailed mechanism in immune abnormality of AE-ILD in IIM patients demands further exploration.

In-hospital IIM patients regularly received immunosuppressive therapy, which greatly increased their vulnerability to bacterial, fungal, or viral infection. More infections, opportunistic bacterial and fungal infections in particular, were hereby identified in this study. Although infectious triggers were found in 10–30% of patients with AE in preceding study (47), no significant association was found between infections and development of AE-ILD after adjusting for disease activity, pulmonary function, medication, etc. In the following intragroup analysis, bacterial infection was found to be associated with unfavorable short-term outcome in DM, PM, or CADM patients complicated with AE-ILD. Similar linkage between infection and short-term outcome was seen in IIM patients (3, 4). And opportunistic infection was as well-recognized as a major cause of mortality in patients with IIM-related ILD (48). However, this is the first study identifying infection as risk factor for unfavorable short-term outcome in patients complicated with AE-ILD.

The mortality rate of patients with AE-ILD was relatively high. For patients with IPF, 46% of deaths are secondary to AE and median survival period after AE is 3-4 months (49). And a high mortality rate (55.6%) was as well-seen in CTD patients with AE-ILD (14). In this study, the short-term mortality rate of AE-ILD group was 39.1%. The relatively high mortality rate of AE-ILD patients indicated much room for improvement in therapeutic regimens. In IIM patients with AE-ILD, a combined use of steroid and DMARDs was found to reduce the short-term mortality rate of these patients. Meanwhile no significant effect was identified in the application of intravenous immunoglobulin. Preceding study revealed a favorable response of exacerbation of ILD in RA patients after receiving a combined therapy of steroid and DMARDs (50). And cyclosporine, tacrolimus, and cyclophosphamide were the major DMARDs used in this study. However, the mostly commonly used DMARD in our study was MMF, the use of which has been proved effective in myositis-related ILD (51). The combined use of steroid and MMF in CTD patients with AE-ILD deserved further exploration in the future. Intravenous immunoglobulin, which was as well-frequently used in patients with ILD or AE-ILD, still played a disputable role in treatment of AE-ILD, especially CTD-related AE-ILD (29, 52). Biologics could be viewed as a two-edge sword in AE-ILD. On the one hand, rituximab, etc. have shown optimistic result in therapy of several AE-ILD cases (52, 53). On the other hand, biologics have also been reported to induce AE-ILD (54, 55). Apart from immunosuppressant treatment, empirical antibiotic therapy is also considered for all patients (56). Application of azithromycin and prophylactic use of co-trimoxazole were found effective in several clinical trials (57–59). Besides, antifibrotic medication, anti-acid therapy, plasma exchange, Polymyxin-B-immobilized fiber column (PMX) and fluid management were as well-found to have potential, yet disputable effect on outcome of AE-ILD patients (29–31).

The most significant limitations of this study are the retrospective and observational nature of the study and the small sample size. Furthermore, absence of records of pulmonary hypertension and several myositis-associated antibodies in over half of the patients also restrained us from figuring out their roles in development of AE-ILD among IIM patients. A large prospective cohort study is essential to confirm our findings and fill in the gaps. In spite of all the limitations, we intended to shed some light on the future study of AE-ILD in patients with DM, PM, or CADM.

## Conclusions

AE-ILD is a fatal complication in IIM patients. Elevated on-admission disease activity, lower DLCO% and diagnosis of CADM were found to be risk factor for development of AE-ILD in patients with DM, PM, or CADM. Speculations on the roles of autoimmune abnormality and hypoxia in development of AE-ILD were hereby brought up. In addition, elevated on-admission disease activity, bacterial infection could be used to predict unfavorable short-term outcome in AE-ILD patients. A therapeutic regimen of steroid and DMARDs was found to reduce short-term death in IIM patients with AE-ILD.

## Data Availability Statement

All datasets generated for this study are included in the article/Supplementary Material.

## Ethics Statement

The studies involving human participants were reviewed and approved by the Institutional Review Board (IRB) of the First Affiliated Hospital of Zhejiang University. The patients/participants provided their written informed consent to participate in this study. Written informed consent was obtained from the individual(s) for the publication of any potentially identifiable images or data included in this article.

## Author Contributions

All authors met the criteria for authorship established by the International Committee of Medical Journal Editors. Specifically, JLia and HC were responsible for substantial contributions to the conception, design, analysis, drafting the work, revising the work, and reviewing of the manuscript. YK, CS, WC, and JLin assisted with the data gathering, revising the work, and reviewing of the manuscript. All the authors listed have approved for publication of the content and have agreed to be accountable for all aspects of the work in ensuring that questions related to the accuracy or integrity of any part of the work.

### Conflict of Interest

The authors declare that the research was conducted in the absence of any commercial or financial relationships that could be construed as a potential conflict of interest.
