# Supplementary material for: Acute Exacerbation of Interstitial Lung Disease in Adult Patients With Idiopathic Inflammatory Myopathies: A Retrospective Case-Control Study
Source: Front Med (Lausanne). 2020 Jan 31;7:12. doi: 10.3389/fmed.2020.00012 (PMC7005087; doi:10.3389/fmed.2020.00012)
Supplement: Supplementary file 1 [file Table_1.docx]

**Supplementary Table 1 Factors aiming at identifying statistically significant factors for development of AE-ILD in IIM patients in univariate logistic regression analysis.**

AE-ILD：Acute exacerbation of interstitial lung disease; IIM: Idiopathic inflammatory myopathies; OR value: Odds ratio value; 95%CI: 95% Confidence interval; y: years; m: months; EBV: Epstein-Barr virus; CMV: Cytomegalo virus; UIP pattern: Usual interstitial pneumonia pattern; MYOACT: Myositis Disease Activity Assessment Visual Analogue Scales; FVC%: Percent-predicted forced vital capacity; TLC: Total lung capacity; FEV1%: Percent-predicted forced expiratory volume in 1 second; FEV1/FVC: Ratio of FEV1 over FVC; DLCO%: Percent-predicted diffusing capacity of the lung for carbon monoxide; ALT: Glutamic pyruvic transaminase; AST: Glutamic oxaloacetic transaminase; Cr: Serum creatinine; LDH: Lactate dehydrogenase; CK: Creatine kinase; CK-MB: Creatine kinase isoenzymes; ANA: Antinuclear antibody; DMARDs: Disease-modifying anti-rheumatic drugs; IVIG: Intravenous immunoglobulin; DM: dermatomyositis; PM: Polymyositis; CADM: Clinically amyopathic dermatomyositis.

| **Factors** | **P value** | **OR** | **95%Cl** |
| --- | --- | --- | --- |
| **Age(y)** | **0.834** | **1.003** | **0.977~1.029** |
| **Sex(male/female)** | **1.000** | **1.000** | **0.541~1.850** |
| **Course of disease(m)** | **0.273** | **0.991** | **0.974~1.007** |
| **Duration of diagnosis delay(m)** | **0.497** | **1.011** | **0.980~1.043** |
| **Clinical manifestations or complications** | | | |
| **Fever** | **0.135** | **1.605** | **0.863~2.988** |
| **Lymphadenectasis** | **0.599** | **1.179** | **0.638~2.181** |
| **Hepatomegaly** | **0.622** | **2.016** | **0.124~32.762** |
| **Splenomegaly** | **0.356** | **1.427** | **0.671~3.035** |
| **Heliotrope rash** | **0.759** | **1.098** | **0.603~2.002** |
| **Gottron’s sign** | **0.475** | **1.246** | **0.682~2.278** |
| **Periungual erythema** | **0.505** | **1.299** | **0.603~2.799** |
| **Mechanic’s hands** | **0.881** | **1.068** | **0.448~2.551** |
| **Raynaud’s phenomenon** | **1.000** | **1.000** | **0.290~3.454** |
| **Muscle pain** | **0.347** | **0.741** | **0.397~1.384** |
| **Muscle weakness** | **0.131** | **0.547** | **0.250~1.196** |
| **Joint pain** | **0.215** | **1.567** | **0.770~3.189** |
| **Joint swelling** | **0.477** | **0.728** | **0.303~1.748** |
| **Dysphagia** | **0.523** | **0.776** | **0.357~1.687** |
| **Dysarthria** | **0.685** | **1.271** | **0.399~4.055** |
| **Respiratory muscle involvement** | **0.475** | **0.558** | **0.112~2.765** |
| **Cardiac involvement** | **0.695** | **1.271** | **0.383~4.222** |
| **Gastrointestinal hemorrhage** | **0.644** | **1.233** | **0.508~2.993** |
| **Bacterial infection** | **0.356** | **1.427** | **0.671~3.035** |
| **Fungal infection** | **0.302** | **1.475** | **0.705~3.087** |
| **Tuberculosis infection** | **0.388** | **2.049** | **0.402~10.452** |
| **EBV or CMV infection** | **0.605** | **0.651** | **0.128~3.318** |
| **Carcinoma** | **0.857** | **1.100** | **0.388~3.123** |
| **UIP pattern** | **0.371** | **0.716** | **0.344~1.490** |
| **Pneumomediastinum** | **0.647** | **1.356** | **0.369~4.986** |
| **On-admission disease activity** | | | |
| **MYOACT score** | **<0.001** | **1.216** | **1.108~1.335** |
| **Pulmonary function test** | | | |
| **FVC%(%)** | **0.681** | **0.996** | **0.978~1.015** |
| **TLC(L)** | **0.214** | **0.810** | **0.582~1.129** |
| **FEV1%(%)** | **0.286** | **0.990** | **0.972~1.008** |
| **FEV1/FVC** | **0.350** | **0.235** | **0.011~4.880** |
| **DLCO%(%)** | **0.010** | **0.974** | **0.955~0.994** |
| **On-admission laboratory findings** | | | |
| **ALT(U/L)** | **0.928** | **1.000** | **0.997~1.003** |
| **AST(U/L)** | **0.615** | **1.000** | **0.998~1.001** |
| **Cr(umol/L)** | **0.968** | **1.000** | **0.997~1.003** |
| **LDH(U/L)** | **0.357** | **1.000** | **0.999~1.000** |
| **CK(U/L)** | **0.200** | **1.000** | **1.000~1.000** |
| **CK-MB(U/L)** | **0.127** | **0.998** | **0.995~1.001** |
| **CRP(mg/L)** | **0.037** | **1.010** | **1.001~1.019** |
| **Ferritin(ng/ml)** | **0.058** | **1.000** | **1.000~1.000** |
| **ANA** | **0.603** | **1.178** | **0.636~2.181** |
| **Comorbidities/Harmful hobbies** | | | |
| **Smoking** | **0.802** | **1.098** | **0.528~2.285** |
| **Alcohol abuse** | **0.593** | **0.802** | **0.358~1.800** |
| **Hypertension** | **0.065** | **1.871** | **0.962~3.636** |
| **Diabetes** | **0.505** | **1.381** | **0.534~3.570** |
| **Hepatitis** | **0.239** | **0.502** | **0.160~1.581** |
| **Allergic History** | **0.058** | **0.340** | **0.111~1.036** |
| **Immunosuppressive therapy** | | | |
| **Steroid monotherapy** | **0.911** | **1.038** | **0.538~2.006** |
| **Steroid+DMARDs** | **0.185** | **0.665** | **0.364~1.216** |
| [**Steroid+IVIG**](http://www.baidu.com/link?url=_srwKTXKnet8GknUvvs0xyTJdpfNOQtIDWHWhe_U5wypEldT9OPh2gCg3LsSDR-5CpyLTLOBAy4p4ov8wle8F6_YWPs4sPX-lyXINgDKaDW) | **0.038** | **2.464** | **1.052~5.770** |
| [**Steroid+DMARDs+IVIG**](http://www.baidu.com/link?url=uciYHxddnq2QF5VJVWJRCy7Q7nEAXlzzmiKvgGzZkrPg72XHW0qrc1acnFRmU-CtSPSZqd_rW-WBKuZFe0OpuS_h9gOsjyItDqvwfb_UtbdGjXJvU0FWCCPVF1qaXYLk) | **0.662** | **0.738** | **0.189~2.881** |
| **IIM subtypes** | | | |
| **DM** | **1.000** | **1.000** | **0.546~1.831** |
| **PM** | **0.124** | **0.584** | **0.295~1.158** |
| **CADM** | **0.038** | **2.464** | **1.052~5.770** |
