# Supplementary material for: Acute Exacerbation of Interstitial Lung Disease in Adult Patients With Idiopathic Inflammatory Myopathies: A Retrospective Case-Control Study
Source: Front Med (Lausanne). 2020 Jan 31;7:12. doi: 10.3389/fmed.2020.00012 (PMC7005087; doi:10.3389/fmed.2020.00012)
Supplement: Supplementary file 2 [file Table_2.docx]

**Supplementary Table 2 Factors aiming at identifying statistically significant factors for unfavorable short-term outcome in IIM patients with AE-ILD in univariate logistic regression analysis**

IIM: Idiopathic inflammatory myopathies; AE-ILD：Acute exacerbation of interstitial lung disease; OR value: Odds ratio value; 95%CI: 95% Confidence interval; y: years; m: months; EBV: Epstein-Barr virus; CMV: Cytomegalo virus; UIP pattern: Usual interstitial pneumonia pattern; MYOACT: Myositis Disease Activity Assessment Visual Analogue Scales; FVC%: Percent-predicted forced vital capacity; TLC: Total lung capacity; FEV1%: Percent-predicted forced expiratory volume in 1 second; FEV1/FVC: Ratio of FEV1 over FVC; DLCO%: Percent-predicted diffusing capacity of the lung for carbon monoxide; ALT: Glutamic pyruvic transaminase; AST: Glutamic oxaloacetic transaminase; Cr: Serum creatinine; LDH: Lactate dehydrogenase; CK: Creatine kinase; CK-MB: Creatine kinase isoenzymes; ANA: Antinuclear antibody; DMARDs: Disease-modifying anti-rheumatic drugs; IVIG: Intravenous immunoglobulin; DM: dermatomyositis; PM: Polymyositis; CADM: Clinically amyopathic dermatomyositis.

| **Factors** | **P value** | **OR** | **95%Cl** |
| --- | --- | --- | --- |
| **Age(y)** | **0.694** | **0.992** | **0.950~1.034** |
| **Sex(male/female)** | **0.243** | **1.846** | **0.660~5.166** |
| **Course of disease(m)** | **0.722** | **0.993** | **0.958~1.030** |
| **Duration of diagnosis delay(m)** | **0.551** | **0.986** | **0.942~1.032** |
| **Clinical manifestations or complications** | | | |
| **Fever** | **0.076** | **2.545** | **0.906~7.151** |
| **Lymphadenectasis** | **0.263** | **0.549** | **0.192~1.569** |
| **Hepatomegaly** | **1.000** | **>1000.000** | **0.000~** |
| **Splenomegaly** | **0.742** | **1.224** | **0.368~4.074** |
| **Heliotrope rash** | **0.648** | **0.791** | **0.289~2.164** |
| **Gottron’s sign** | **0.289** | **0.577** | **0.209~1.593** |
| **Periungual erythema** | **0.494** | **0.635** | **0.172~2.337** |
| **Mechanic’s hands** | **0.722** | **1.295** | **0.312~5.374** |
| **Raynaud’s phenomenon** | **0.999** | **<0.001** | **0.000~** |
| **Muscle pain** | **0.197** | **2.000** | **0.697~5.736** |
| **Muscle weakness** | **0.772** | **1.200** | **0.350~4.109** |
| **Joint pain** | **0.835** | **1.128** | **0.364~3.494** |
| **Joint swelling** | **0.923** | **0.927** | **0.201~4.276** |
| **Dysphagia** | **0.019** | **5.647** | **1.329~23.998** |
| **Dysarthria** | **0.085** | **7.238** | **0.759~69.030** |
| **Respiratory muscle involvement** | **0.749** | **1.583** | **0.095~26.524** |
| **Cardiac involvement** | **0.165** | **0.193** | **0.019~1.970** |
| **Gastrointestinal hemorrhage** | **0.282** | **2.187** | **0.526~9.095** |
| **Bacterial infection** | **0.002** | **9.429** | **2.284~38.930** |
| **Fungal infection** | **0.064** | **3.094** | **0.938~10.201** |
| **Tuberculosis infection** | **0.999** | **<0.001** | **0.000~** |
| **EBV or CMV infection** | **0.999** | **<0.001** | **0.000~** |
| **Carcinoma** | **0.999** | **<0.001** | **0.000~** |
| **UIP pattern** | **0.604** | **1.379** | **0.409~4.650** |
| **Pneumomediastinum** | **0.165** | **5.182** | **0.508~52.902** |
| **On-admission disease activity** | | | |
| **MYOACT score** | **0.012** | **1.247** | **1.050~1.481** |
| **Pulmonary function test** | | | |
| **FVC%(%)** | **0.199** | **0.979** | **0.947~1.011** |
| **TLC(L)** | **0.981** | **0.994** | **0.601~1.643** |
| **FEV1%(%)** | **0.227** | **0.976** | **0.939~1.015** |
| **FEV1/FVC** | **0.478** | **0.169** | **0.001~23.014** |
| **DLCO%(%)** | **0.750** | **0.994** | **0.957~1.032** |
| **On-admission laboratory findings** | | | |
| **ALT(U/L)** | **0.502** | **0.998** | **0.994~1.003** |
| **AST(U/L)** | **0.172** | **0.997** | **0.993~1.001** |
| **Cr(umol/L)** | **0.067** | **1.020** | **0.999~1.041** |
| **LDH(U/L)** | **0.674** | **1.000** | **0.998~1.001** |
| **CK(U/L)** | **0.179** | **1.000** | **1.000~1.000** |
| **CK-MB(U/L)** | **0.192** | **0.994** | **0.984~1.003** |
| **CRP(mg/L)** | **0.223** | **1.008** | **0.995~1.022** |
| **Ferritin(ng/ml)** | **0.298** | **1.000** | **1.000~1.000** |
| **ANA** | **0.058** | **0.363** | **0.127~1.036** |
| **Comorbidities/Harmful hobbies** | | | |
| **Smoking** | **0.742** | **1.224** | **0.368~4.074** |
| **Alcohol abuse** | **0.947** | **1.048** | **0.264~4.157** |
| **Hypertension** | **0.020** | **3.611** | **1.224~10.654** |
| **Diabetes** | **0.501** | **1.667** | **0.376~7.379** |
| **Hepatitis** | **0.646** | **1.609** | **0.212~12.223** |
| **Allergic History** | **0.646** | **1.609** | **0.212~12.223** |
| **Immunosuppressive therapy** | | | |
| **Steroid monotherapy** | **0.746** | **1.198** | **0.402~3.570** |
| **Steroid+DMARDs** | **0.002** | **0.156** | **0.048~0.505** |
| [**Steroid+IVIG**](http://www.baidu.com/link?url=_srwKTXKnet8GknUvvs0xyTJdpfNOQtIDWHWhe_U5wypEldT9OPh2gCg3LsSDR-5CpyLTLOBAy4p4ov8wle8F6_YWPs4sPX-lyXINgDKaDW) | **0.018** | **4.922** | **1.317~18.387** |
| [**Steroid+DMARDs+IVIG**](http://www.baidu.com/link?url=uciYHxddnq2QF5VJVWJRCy7Q7nEAXlzzmiKvgGzZkrPg72XHW0qrc1acnFRmU-CtSPSZqd_rW-WBKuZFe0OpuS_h9gOsjyItDqvwfb_UtbdGjXJvU0FWCCPVF1qaXYLk) | **0.999** | **>1000.000** | **0.000~** |
| **IIM subtypes** | | | |
| **DM** | **0.974** | **0.983** | **0.357~2.706** |
| **PM** | **0.492** | **1.507** | **0.468~4.850** |
| **CADM** | **0.494** | **0.635** | **0.172~2.337** |
