# Supplementary material for: Acute Exacerbation of Interstitial Lung Disease in Adult Patients With Idiopathic Inflammatory Myopathies: A Retrospective Case-Control Study
Source: Front Med (Lausanne). 2020 Jan 31;7:12. doi: 10.3389/fmed.2020.00012 (PMC7005087; doi:10.3389/fmed.2020.00012)
Supplement: Supplementary file 4 [file Data_Sheet_2.docx]

**Supplementary Data 2 Details on infection in the matched control group.**

There existed 43 cases with infection in the matched control group. 14 had bacterial infection, 14 had fungal infection, 3 were diagnosed with tuberculosis, 2 suffered from EBV infection, one was infected with CMV, 6 had both bacterial and fungal infection, one had both bacterial and CMV infection and 2 suffered from both fungal and EBV infection. Bacterial infection included 5 cases with Escherichia coli, 3 cases of Acinetobacter baumannii, 3 cases with Klebsiella pneumonia, 3 cases with Pseudomonas aeruginosa, 2 cases with Staphylococcus aureus, one case with Stenotrophomonas maltophilia, one case with Enterobacter cloacae, one case with Listeria monocytogenes, one case with Haemophlus influenza and one case with Streptococcus viridans. And fungal infection included 17 cases with medium to large amount of Candida albicans, 3 cases with Cryptococcus, one case with Aspergillus fumigatus and one case with large amount of Candida glabrata. Bacterial infection (48.8%) and fungal infection (51.2%) were hereby the most common infections in the matched control group. Only 11 patients (5 with bacterial infection, 5 with fungal infection, one with both bacterial and fungal infection) in the control group were identified based on positive result of BALF culture.
